# Liver metabolomics reveals potential mechanism of Jieduan-Niwan formula against acute-on-chronic liver failure (ACLF) by improving mitochondrial damage and TCA cycle

**DOI:** 10.1186/s13020-023-00858-x

**Published:** 2023-11-30

**Authors:** Jiajun Liang, Xiaoyi Wei, Weixin Hou, Hanjing Wang, Ruimin Ma, Yanbin Gao, Yuqiong Du, Qiuyun Zhang

**Affiliations:** 1 School of Traditional Chinese Medicine, Capital Medical University, Beijing, 100069 China; 2 Beijing Key Laboratory of TCM Collateral Disease Theory Research, Beijing, 100069 China; 3 Beijing Friendship Hospital, Capital Medical University, Beijing, 100050 China; 4 Department of Gastroenterology, Peking Union Medical College Hospital, Peking Union Medical College, Chinese Academy of Medical Sciences, Beijing, 100730 China

**Keywords:** Acute-on-chronic liver failure (ACLF), Jieduan-Niwan formula (JDNWF), Metabolomics, TCA cycle, Energy metabolism, Mitochondrial quality control

## Abstract

**Background:**

Acute-on-chronic liver failure (ACLF) is a refractory disease with high mortality, which is characterized by a pathophysiological process of inflammation-related dysfunction of energy metabolism. Jieduan-Niwan formula (JDNWF) is a eutherapeutic Chinese medicine formula for ACLF. However, the intrinsic mechanism of its anti-ACLF effect still need to be studied systematically.

**Purpose:**

This study aimed to investigate the mechanism of JDNWF against ACLF based on altered substance metabolic profile in ACLF the expression levels of related molecules.

**Materials and methods:**

The chemical characteristics of JDNWF were characterized using ultra performance liquid chromatography (UPLC) coupled with triple quadrupole mass spectrometry. Wistar rats subjected to a long-term CCL_4_ stimulation followed by a combination of an acute attack with LPS/D-GalN were used to establish the ACLF model. Liver metabolites were analyzed by LC–MS/MS and multivariate analysis. Liver function, coagulation function, histopathology, mitochondrial metabolic enzyme activity and mitochondrial damage markers were evaluated. The protein expression of mitochondrial quality control (MQC) was investigated by western blot.

**Results:**

Liver function, coagulation function, inflammation, oxidative stress and mitochondrial enzyme activity were significantly improved by JDNWF. 108 metabolites are considered as biomarkers of JDNWF in treating ACLF, which were closely related to TCA cycle. It was further suggested that JDNWF alleviated mitochondrial damage and MQC may be potential mechanism of JDNWF improving mitochondrial function.

**Conclusions:**

Metabolomics revealed that TCA cycle was impaired in ACLF rats, and JDNWF had a regulatory effect on it. The potential mechanism may be improving the mitochondrial function through MQC pathway, thus restoring energy metabolism.

**Supplementary Information:**

The online version contains supplementary material available at 10.1186/s13020-023-00858-x.

## Introduction

Acute-on-chronic liver failure (ACLF) is a clinical syndrome characterized by high short-term mortality in patients with acute or subacute decompensation of liver function over a short period based on previous chronic liver disease, which may be accompanied by extrahepatic organ failure. This life-threatening and complex disease is aggressive and seriously endangers human health [1, 2]. In Europe and the United States, the main trigger of ACLF is a liver injury caused by drugs and alcohol, while in the Asia Pacific it is mainly caused by hepatitis B virus (HBV) infection. The 28-day and 90-day mortality rates for patients with ACLF under the Asia–Pacific definition have been reported to be 41.9% and 56.1%, respectively [1]. Therefore, it is urgent to propose an effective therapeutic strategy for ACLF.

Tradition Chinese medicine (TCM), with its multi-pathway and multi-target features, is widely applied in the prevention and cure of liver diseases in China. Jieduan-Niwan formula (JDNWF) is an empirical formula for ACLF in China. The therapeutic principle and method of JDNWF combines the “Jieduan method” and “Niwan method” derived from the “Treatise on Epidemic Febrile Diseases” and “Yu Yi Cao” in the Qing Dynasty and recommended by the hepatobiliary disease branch of the Chinese Society of Traditional Chinese Medicine [3]. According to TCM theory, the pathogenesis of ACLF is “toxicity damages the liver body”, while JDNWF has the effect of heat-clearing and detoxifying, jaundice-treating, blood stasis-removing, liver, and kidney-nourishing as well as Yin and Yang-regulating. Our previous study confirmed the clinical efficacy of JDNWF against ACLF, by reducing total bilirubin (TBiL) and increasing prothrombin activity (PTA) in patients [4, 5], and found that this formula has anti-apoptotic effects in hepatocytes [6–8]. However, the underlying mechanisms still need to be further explored.

Metabolomics is a widely used method to discover biomarkers by detecting changes of metabolites in biological fluids, tissues, etc. [9, 10]. Metabolomics can depict the metabolic status and reveal the metabolic pathways of the organism after pathological stimuli and drug effects, which is an essential means to understand the holistic therapeutic effect of TCM [11]. Currently, metabolomics has been widely applied to TCM-related studies and has made a significant contribution to investigating the mechanism of TCM in preventing and treating various diseases [12–15]. Therefore, in this study, untargeted metabolomics was employed to investigate the potential mechanism of JDNWF against ACLF, and we further detected the protein expression levels of related molecular pathways.

## Materials and methods

### Preparation of JDNWF

JDNWF consists of ten herbs including *Phyllanthus amarus* Schumach. & Thonn, *Trichosanthes kirilowii* Maxim, *Lysimachia christinae* Hance, *Astragalus mongholicus* Bunge, *Viscum coloratum* (Kom.) Nakai, *Panax notoginseng* (Burkill) F.H.Chen, *Curcuma phaeocaulis* Valeton, *Salvia miltiorrhiza* Bunge, *Rehmannia glutinosa* (Gaertn.) DC and *Aconitum carmichaelii* Debeaux (Table 1). These 10 herbs were purchased from Beijing TongRenTang (Group) Co. Ltd., Beijing, China and mixed in the proportions previously described [16]. After soaking in distilled water for 4 h, *Aconitum carmichaelii* Debeaux. was boiled for 30 min first, followed by the remaining herbs being boiled together for 45 min. The liquid collected and the herbal mixture was boiled again for 30 min with distilled water. The two aqueous decoctions were mixed, filtered and subsequently concentrated. Finally, the decoction was concentrated at 4.34 g/ml for rat administration.

**Table 1:** The 10 botanical plant of JDNWF

Chinese Name	Botanical Name	Genus	Family	Weight (g)	Part used
Ku Wei Ye Xia Zhu	*Phyllanthus amarus* Schumach. & Thonn	*Phyllanthus*	*Phyllanthaceae*	30	Herb
Gua Lou	*Trichosanthes kirilowii* Maxim	*Trichosanthes*	*Cucurbitaceae*	30	Fruit
Jin Qian Cao	*Lysimachia christinae* Hance	Lysimachia	*Primulaceae*	30	Herb
Huang Qi	*Astragalus mongholicus* Bunge	Astragalus	*Fabaceae*	30	Root
Hu Ji Sheng	*Viscum coloratum* (Kom.) Nakai	Viscum	*Santalaceae*	30	Stem, leaf
San Qi	*Panax notoginseng* (Burkill) F.H.Chen	Panax	*Araliaceae*	6	Root
E Zhu	*Curcuma phaeocaulis* Valeton	Curcuma	*Zingiberaceae*	6	Root
Dan Shen	*Salvia miltiorrhiza* Bunge	Salvia	*Lamiaceae*	20	Root
Di Huang	*Rehmannia glutinosa* (Gaertn.) DC	Rehmannia	*Orobanchaceae*	20	Root
Fu Zi	*Aconitum carmichaelii* Debeaux	Aconitum	*Ranunculaceae*	15	Root

^*^The accepted name of plant has been checked with http://mpns.kew.org (2022.11.24)

### Identification of compounds in JDNWF by UPLC- MS/MS

For quality control of JDNWF, samples were freeze-dried and ground into powder for UPLC-MS/MS analysis (UPLC, SHIMADZU Nexera X2; MS, Applied Biosystems 4500 QTRAP). An Agilent SB-C18 column (1.8 µm, 2.1 mm*100 mm) was employed and the mobile phases were pure water with 0.1% formic acid (A) and acetonitrile with 0.1% formic acid (B). The gradient elution degree was set as 5% B phase at 0 min and linearly increased to 95% (B) within 9 min, which was kept for 1 min. From 10 to 11 min, 5% (B) was adjusted and balanced at 5% to 14 min. The flow rate was 0.35 ml/min and 4 μl filtrate was injected for analysis with a column temperature at 40 °C. The positive and negative ion modes are controlled by Analyst 1.6.3 software (AB Sciex). Operating parameters of ESI source are as follows: ion source, turbine spray; Source temperature 550 °C; Ion spray voltage (IS) 5500 V (positive ion mode)/− 4500 V (negative ion mode); The ion source gas I (GSI), gas II (GSII) and curtain gas (CUR) are set to 50 psi, 60 psi and 25.0 psi respectively, and the collision-induced ionization parameter is set to high. Instrument tuning and quality calibration were performed with 10 and 100 μmol/l polypropylene glycol solution in triple quadrupole (QQQ) and LIT modes, respectively. QQQ scan uses MRM mode and sets collision gas (nitrogen) to medium. DP and CE of each MRM ion pair were completed by further DP and CE optimization. A specific set of MRM ion pairs was monitored at each period based on the eluted metabolites in each period. Compounds in JDNWF were identified, including quercetin, kaempferol, catalpol, notoginsenoside R1, gallic acid, tanshinone IIA, tanshinone I, cryptotanshinone, formononetin and luteolin. Chemical mass spectra of JDNWF and information of major components were shown in Additional file 1: Figure S1 and Table S1.

### Animal and models

**Fig. 1:**
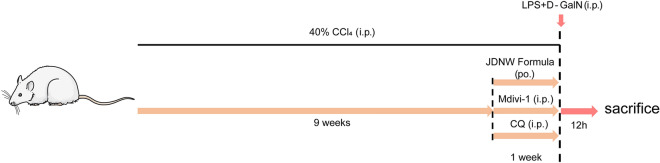
Experimental procedures of model establishment

Wistar male rats, weighing 180–220 g, were purchased from Beijing Vital River Laboratory Animal Technology Co., Ltd., Beijing, China. The animal experiments were carried out in the Experimental Animal Center of Capital Medical University. All rats were fed and watered freely in a specified-pathogens free (SPF) environment at 20–25 ℃, 50% humidity and 12 h-light–dark cycle. The design and performing of animal experiments are approved by the Animal Experimentation and Laboratory Animal Welfare Committee of Capital Medical University. Rats were randomly divided into five groups: normal control group (NC, n = 9), ACLF model group (ACLF, n = 9), JDNWF treatment group (JDNWF, n = 9), mdivi-1 treatment group (mdivi-1) and chloroquine treatment group (CQ). Rats were fed adaptively for 7 days before the experiment. ACLF rat model was established according to previous reports (Fig. 1) [7]: Rats in the ACLF group and JDNWF group were injected intraperitoneally with 40% carbon tetrachloride (CCl_4_) olive oil solution (1.5 ml/kg) twice a week. After ten weeks, acute liver injury was induced by intraperitoneal injection of LPS (100 μg/kg) and D-GalN (400 mg/kg). Rats in the NC group were given equal doses of saline.

A week before the induction of acute liver injury, JDNWF group was given 21.7 g/kg JDNWF decoction for intragastric administration according to our previous studies [17]. Mdivi-1 group was intragastrically injected with mdivi-1 (1 mg/k) [18, 19] and CQ group was intragastrically injected with CQ (20 mg/kg) [20].Rats in each group were sacrificed 12 h after the acute attack. Before tissue collection, rats were anesthetized with 1% sodium pentobarbital (40 mg/kg) injected intraperitoneally. After rat was deeply anesthetized, blood was collected from the abdominal aorta and left to coagulate at room temperature for 4 h, followed by centrifugation at 4000 rpm for 15 min at 4 °C to obtain serum samples. The rat livers were completely removed and quickly rinsed in PBS. A fraction of the liver was fixed using 4% paraformaldehyde for histopathological observation and the remaining liver was stored at − 80 °C after rapid freezing in liquid nitrogen. The rat cervical vertebrae were dislocated for euthanasia.

### Liver function assessment

The levels of serum liver function indicators alanine aminotransferase (ALT), aspartate aminotransferase (AST), total bilirubin (TBIL), and albumin (ALB) were detected using a Hitachi 7600 automatic biochemistry analyzer (Hitachi, Tokyo, Japan).

### Coagulation function assay

Activated partial thromboplastin time (APTT), prothrombin time (PT), international normalized ratio (INR) and Fibrinogen (FIB) levels were measured in rat plasma treated with Na-citrate using a Beckman Coulter ACL-TOP 700 coagulation analyzer (Beckman/Instrumentation Laboratory, Florida, USA).

### Histological examination

The liver tissue fixed with paraformaldehyde was embedded in paraffin and then stained with hematoxylin–eosin (HE) and Masson. The stained samples were scanned by Pannoramic SCAN (3D HISTECH) to obtain images.

### Ultrastucture observation

Samples for observation by transmission electron microscope (TEM) were processed as in previous studies [7] and the mitochondrial structure was observed using a Hitachi 7700 TEM (Hitachi, Tokyo, Japan).

### Non-targeted metabolomics analysis

#### Sample preparation and quality control

A 100 mg sample of tissue ground in liquid nitrogen was vortexed and shaken with 500 μl of 80% methanol in water, left to stand for 5 min in an ice bath, and centrifuged at 15,000*g* for 20 min at 4 °C. The methanol concentration was diluted to 53% with mass spectrometry grade water. The supernatant was collected by centrifugation at 15000*g* for 20 min at 4 °C and analyzed by LC–MS. The Q-Exactive HF-X Mass Spectrometer was connected in tandem with Vanquish UHPLC system. To ensure the stability and reproducibility of the instrumental analysis, quality control (QC) samples were prepared by mixing equal amounts of each sample. Three QC samples were inserted to balance the LC–MS system before testing the sample. After that, four QC samples were inserted during the sample testing to evaluate the stability of the system. Besides, blank samples were set for excluding background ions. Finally, PCA analysis was used to visualize the testing samples and QC samples, and showed an aggregation of QC sample points, suggesting no signal drift. Besides, PC1 values of all sample were also used to assess whether the laboratory sample is outlier and sample points outside the control limit (mean ± 3SD) are considered outliers. As shown in Additional file 1: Figure S2, most of the points were distributed within mean ± 2SD (Additional file 1: Figure S2).

#### LC–MS/MS analysis

All samples were analyzed in both positive and negative mode. The spectra were performed on a Hypesil Gold column (C18) (100 mm × 2.1 mm, 1.9 μm). The mobile phase in the positive mode consisted of 0.1% formic acid (A) and methanol (B) in the positive mode and 5 mM ammonium acetate (A) and methanol (B) in the negative mode. The gradient elution times were 0 min, 98% (A), 2% (B); 1.5 min, 98% (A), 2% (B); 12 min, 0% (A), 100% (B); 14 min, 0% (A), 100% (B); 14.1 min, 98% (A), 2% (B); 17 min, 98% (A), 2% (B). Scanning range 100–1500 m/z. ESI source Settings are as follows: Spray Voltage:3.2 kV; Sheath gas flow rate:40 arb; Aux Gasflow rate:10 arb; For Capillary Temp:320 °C. MS/MS secondary scanning is data-dependent scan.

#### Data processing

The chromatograms obtained from LC/MS analysis were imported into Compound Discoverer 3.1 (ThermoFisher Scientific, USA) for processing and filtering based on retention time, mass-to-charge ratio, and other parameters. The peak alignment parameters for each sample are set to a retention time deviation of 0.2 min and a mass deviation of 5 ppm. Peak extraction was then performed based on a mass deviation of 5 ppm, a signal intensity deviation of 30%, a signal-to-noise ratio of 3, minimum signal intensity, and summed ions and compared with mzCloud (https://www.mzcloud.org/), mzVault and Masslist databases. A mass tolerance range of 5 ppm was set for matching the compounds we assayed to the metabolite information in the database. According to the mzCloud database, if the mass difference between the two parent ions is well within the mass tolerance, the test compound was considered as full match to that metabolite in the database. Otherwise, a further prediction based on the mzVault and MassList databases was perform to compare the daughter ion fragments. After the above steps, a data normalization was carried out on the peak areas of the metabolites. Firstly, the abundance of all features in each sample was divided by the median abundance of that sample to correct the sample library size. Then, log transformations were performed on all metabolite contents to correct the content matrix to bring the metabolite content distribution close to normality. Finally, the abundance of all samples corresponding to the feature was subtracted from the mean of that feature abundance and divided by the standard deviation of that feature so that the mean and standard deviation of all metabolites were at the same level. After normalization, the median and upper and lower quartiles of metabolite content were essentially at the same level.

#### Multivariate analysis and metabolic pathways enrichment

The processed metabolite data were uploaded to Wekemo Bioincloud (https://bioincloud.tech/) for further analysis. Enrichment analysis of liver differential metabolites in each group of rats was performed with Over-Representation Analysis (ORA) provided on the website. Differences in metabolic patterns between groups were identified by principal component analysis (PCA), orthogonal partial least squares discriminant analysis (OPLS-DA). Differences in various metabolites between groups were compared using a two-sided t-test, and differential metabolites were screened in the OPLS-DA model based on parameters of P < 0.05, VIP > 1. Finally, the marker metabolites screened based on OPLS-DA model were uploaded to MetaboAnalyst 5.0 (https://www.metaboanalyst.ca/faces/home.xhtml) for pathway analysis.

### Liver ATP and serum GDH assay

Frozen liver in frozen double-distilled water to make a 10% homogenate. Liver ATP levels were assayed according to the instructions provided in the kit (Nanjing Jiancheng Bioengineering Insitute, Nanjing, China). The serum obtained was used to detect GDH activity, according to the instructions provided with the kit (Nanjing Jiancheng Bioengineering Insitute, Nanjing, China).

### Energy metabolism markers lactate and NAD + /NADH assay

The serum obtained was used to detect lactate, according to the instructions provided with the kit (Nanjing Jiancheng Bioengineering Institute, Nanjing, China). Frozen liver in frozen double-distilled water to make a 10% homogenate. Liver NAD + /NADH ratio was assayed according to the instructions provided in the kit (Beyotime, Shanghai, China).

### Mitochondrial membrane potential analysis

Fluorescent probe JC-10 (4A Biotech, Beijing, China) was used to estimate mitochondrial membrane potential (MMP). Single cell suspension was prepared from fresh rat liver and incubated with 20 μM JC-10 solution for 30 min in dark light according to the kit instructions. Finally, fluorescence changes of Ex/Em = 490/525 and 490/590 were detected by FL1 and FL2 channels with NovoCyte 3130 (ACEA Biosciences, USA).

### Markers of oxidative stress (MDA, SOD and GSH) assay

Liver tissue homogenates were prepared for MDA, SOD and GSH assays according to the instructions of the kit (Beyotime, Shanghai, China).

### Activity of metabolic enzymes involved in the TCA cycle

Detection of hepatic alpha-ketoglutarate dehydrogenase (α-KGDH), fumarase (FUM), cytosolic isocitrate dehydrogenase (ICDHc), succinate dehydrogenase (SDH), cis-aconitate dehydrogenase (ACO), and mitochondrial isocitrate dehydrogenase activity (ICDHm) in rats according to the kit instructions (Kang Jia Hong Yuan Biological Technology Co., Ltd, Beijing, China).

### The level of inflammatory factors TNF-α and IL-6

The levels of liver inflammatory factor TNF-αand IL-6 were measured in each group according to the ELISA kit instructions (Kang Jia Hong Yuan Biological Technology Co., Ltd, Beijing, China).

### Western blot analysis

The liver tissue protein samples (3 μg/μl) were collected as previously described and subsequently separated by SDS polyacrylamide gel electrophoresis (SDS-PAGE) and transferred to PVDF membranes. After blocking with 5% skimmed milk powder, the bands were incubated overnight at 4 °C with the appropriate primary antibodies against PGC-1α (1:3000, Proteintech, USA), NRF1 (1:1000, Cell Signaling Technology, USA), TFAM (1:1000, Proteintech, USA), MFN2 (1:1000, Cell Signaling Technology, USA), DRP1 (1:1000, Cell Signaling Technology, USA), PINK1 (1:1000, Proteintech, USA), Parkin (1:1000, Cell Signaling Technology, USA), LC3B (1:500, Genetex, USA), P62 (1:200, Santa Cruz, USA), and β-actin (1:1000, Cell Signaling Technology, USA). Bands were washed with TBST and incubated with the corresponding secondary antibody, including Donkey Anti-Mouse IgG (Proteintech, USA) and Goat Anti-Rabbit IgG (Lablead, China), for 1 h at room temperature. Protein expression was observed using a Vilber FUSION FX6 XT gel chemiluminescence imaging analysis system (Vilber Lourmat, Marne La Vallee, France). The images were analyzed semi-quantitatively using ImageJ software. Results are presented as the ratio of the target protein to reference protein.

### Statistical analysis

Metabolomics-related data were analyzed as described above. Other data were analyzed using GraphPad software (Prism 7.00). For comparison of two groups, the t-test was used for data with equal variance, otherwise, the Mann–Whitney test was used; for comparison of multiple groups, the one-way ANOVA was used for data with equal variance, otherwise, the Kruskal-Walli’s test was used. P < 0.05 were statistically significant.

## Results

### Hepatic injury in ACLF rats was attenuated by JDNWF

**Fig. 2:**
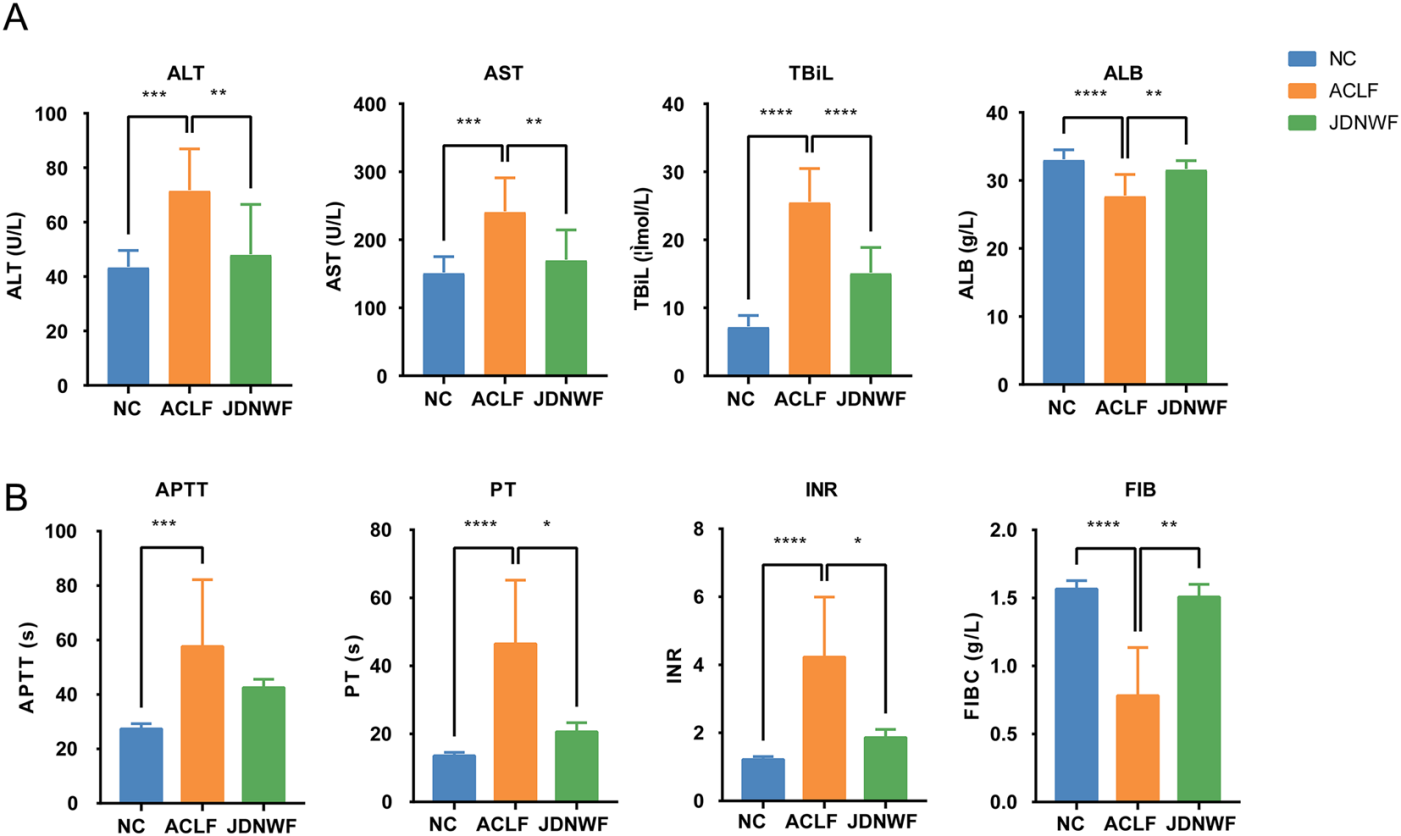
Effort of JDNWF on improving liver damage in ACLF rat. JDNWF improves liver function pa-rameters ALT, AST, TBiL and ALB (**A**) and coagulation parameters APTT, PT, INR and FIB (**B**) in ACLF rats. (*P < 0.05, **P < 0.01, ***P < 0.001, ****P < 0.0001)

**Fig. 3:**
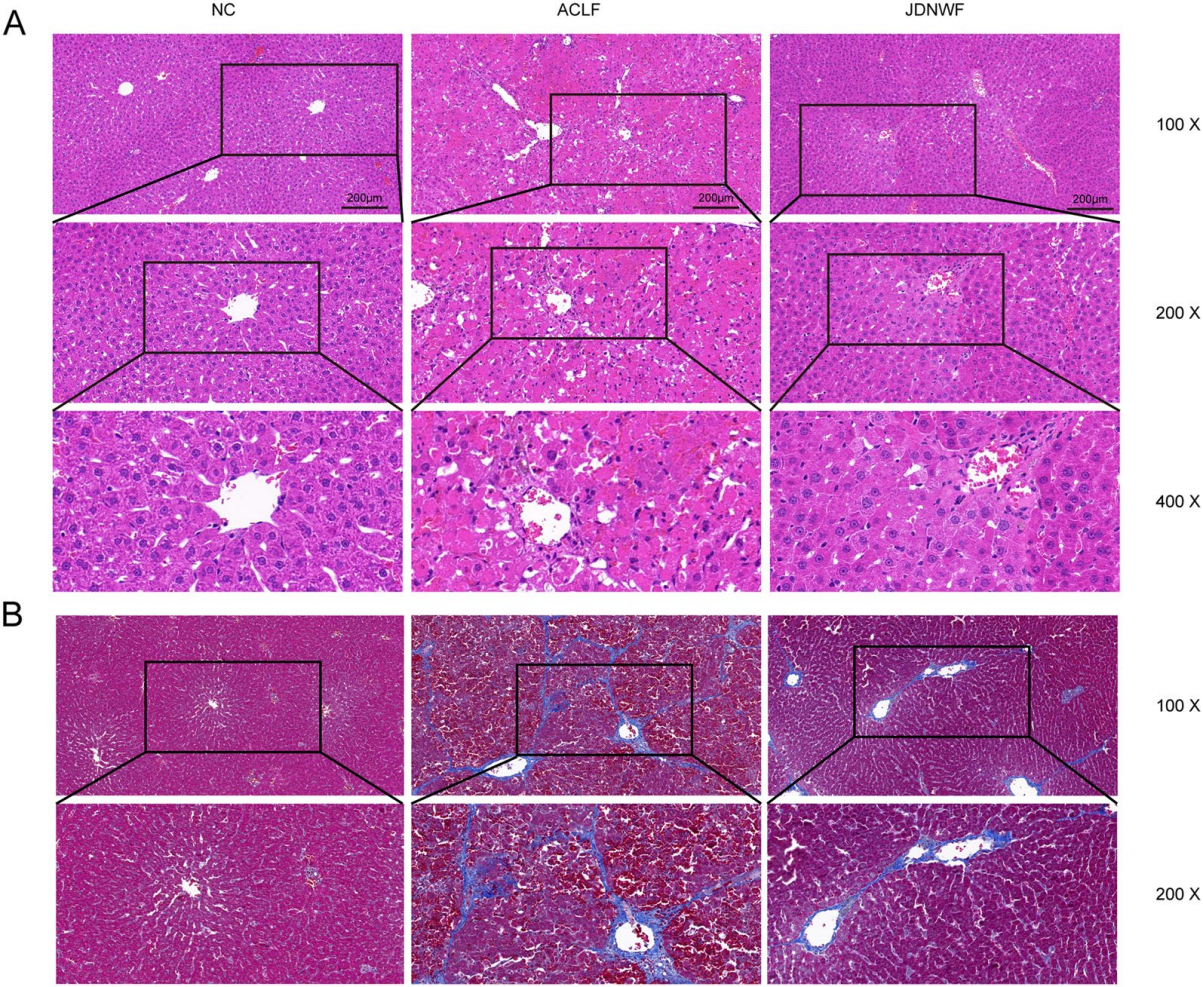
Liver pathological damage of rats in HE (**A**) and Masson (**B**) staining

To investigate the effect of JDNWF against ACLF, the ACLF rat model was constructed by intraperitoneal injection with CCL_4_ to induce cirrhosis in rats, with a subsequent acute hit using LPS/D-GalN, and treated with JDNWF. We first evaluated the liver function of rats in each group. Compared with the NC group, rats in the ACLF group had significantly higher levels of AST, ALT, and TBIL, significantly lower ALB (Fig. 2A). However, serum ALT, AST and TBIL levels were significantly decreased after JDNWF treatment. Coagulation function is also one of the key indicators to evaluate the hepatic synthetic function and lower FIB, prolonged APTT and PT, and increased INR were observed in ACLF rats. As expected, JDNWF treatment was contribute to the restoration of coagulation function (Fig. 2B). Subsequently, the histopathological changes in rat liver were observed by HE and Masson staining, and it was found that the hepatocytes in the NC group were polygonal in shape and arranged radially with the central vein as the center, and no obvious proliferating fibers were observed; The livers of rats in the ACLF group showed structural destruction of liver lobules, large and sub-large foci of necrosis, infiltration of inflammatory cells, congestion of the hepatic sinusoids, and bridging of the confluent areas with proliferating collagen fibers, which formed a pseudobulbar structure. In contrast, the disordered liver structure of rats in the JDNWF group was improved (Fig. 3A, B). The results above suggested that ACLF rats suffered from severe liver pathological damage and dysfunction of liver function and coagulation, while JDNWF could effectively improve the liver injury in ACLF model rats.

### Liver metabolite analysis and multiple statistical analysis

**Fig. 4:**
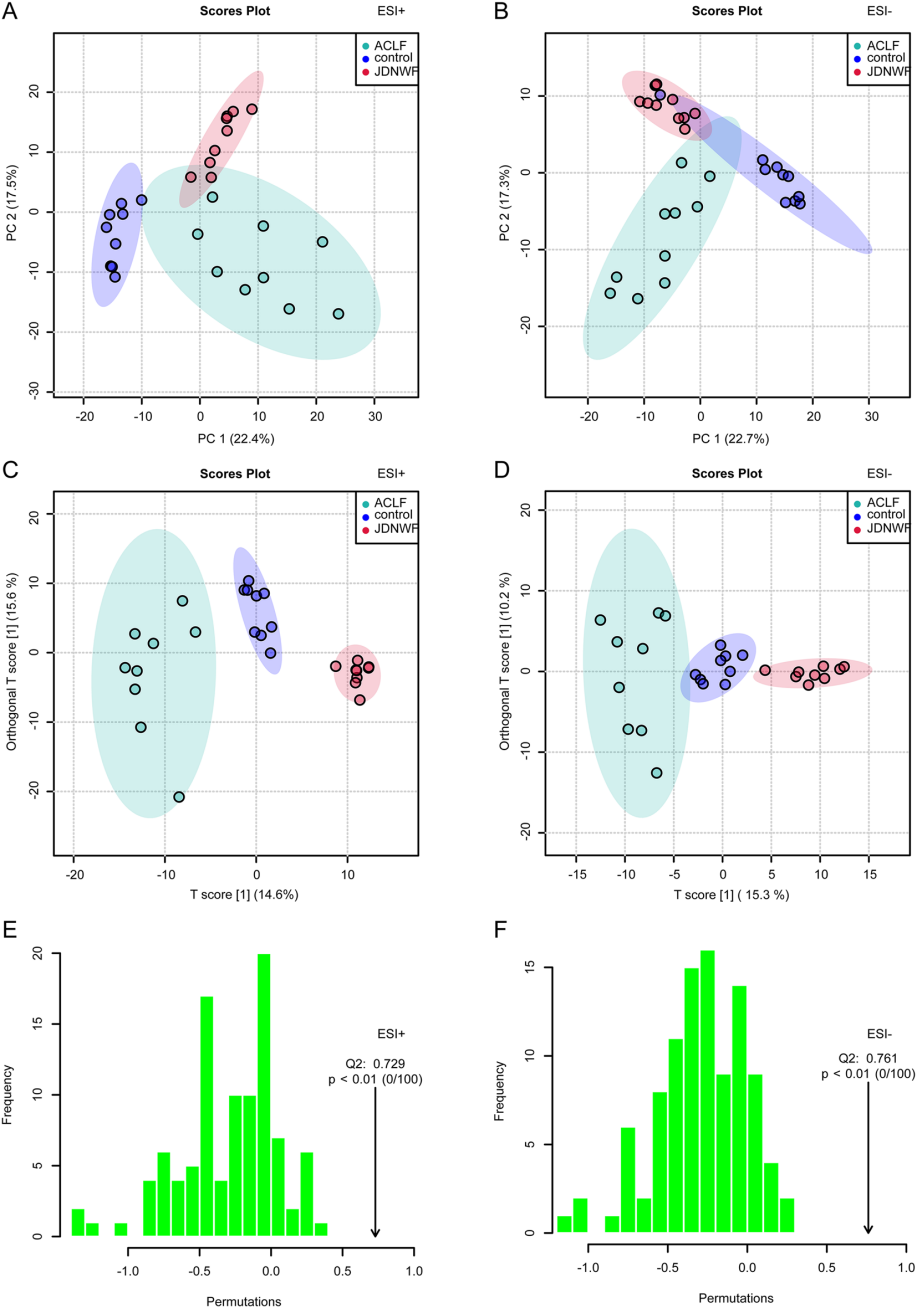
PCA and OPLS-DA score plot of rats’ liver sample. PCA plot in positive mode (**A**) and negative mode (**B**); OPLS-DA plot and permutations test in positive mode (**C**, **E**) and negative mode (**D**, **F**)

In this study, LC–MS/MS approach was applied to investigate the metabolite changes in liver tissues of rats in each group, and the metabolic profiles of liver tissues of rats in each group were generated. The representative TIC diagrams were shown as Additional file 1: Figure S3. Based on the metabolite chromatogram of positive and negative ESI modes, we established unsupervised PCA and supervised OPLS-DA models to visualize the metabolic pattern between rats. Both in positive and negative mode, PCA plots showed a significant trend of separation between the NC, ACLF and JDNWF groups (Fig. 4A, B). Similarly, the parallel performance in the OPLS-DA plot (ESI + : R2X = 0.438, R2Y = 0.965, Q2 = 0.729; ESI + : R2X = 0.255, R2Y = 0.928, Q2 = 0.761) indicates the successful establishment of the ACLF rat model and supports the efficacy of JDNWF treatment (Fig. 4C–F).

### Biomarker screening and metabolic pathway analysis

**Fig. 5:**
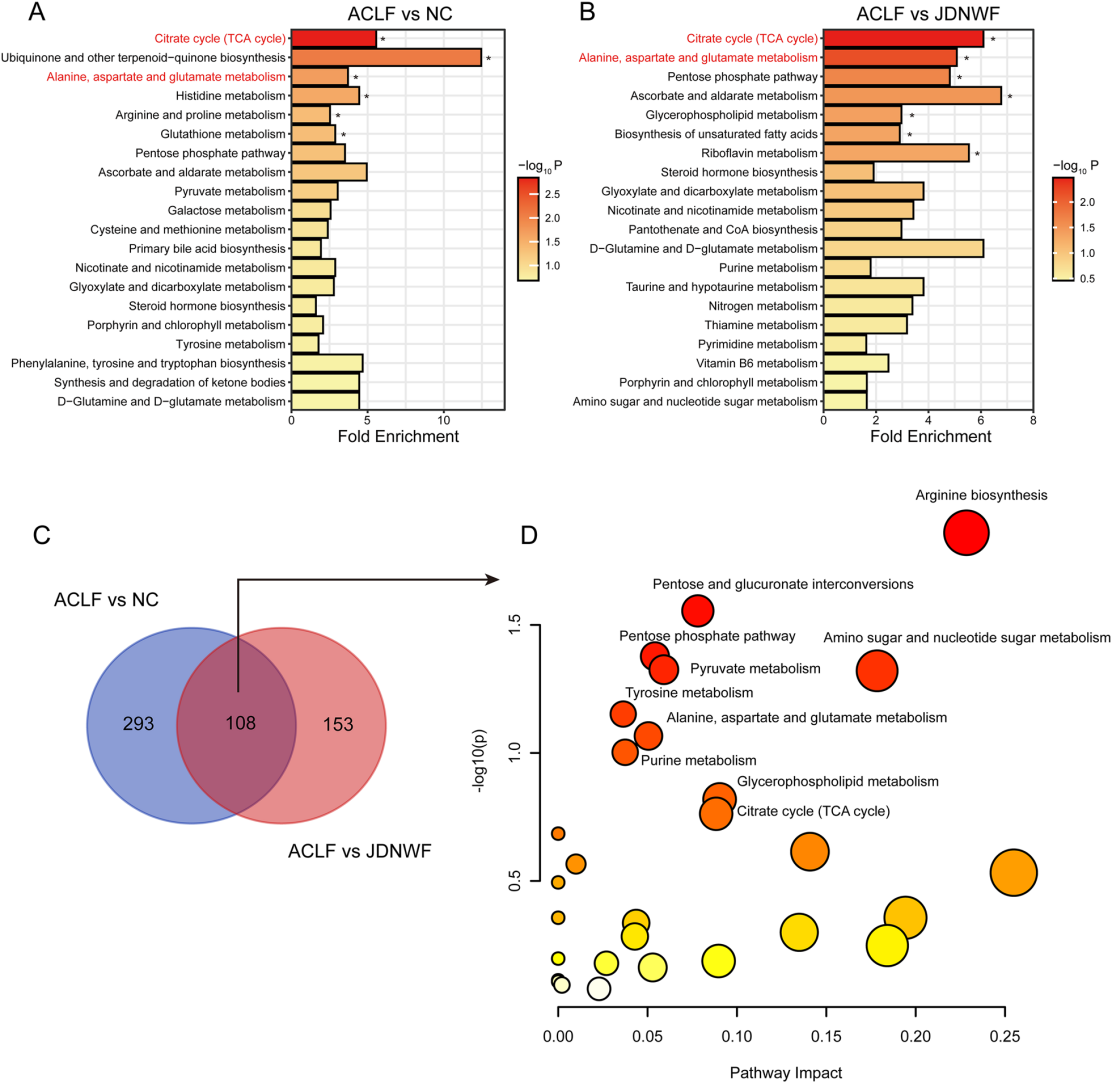
Metabolites and metabolic pathways significantly altered by JDNWF intervention in ACLF rat. Top 20 metabolic pathway associated with ACLF vs NC (**A**) and ACLF vs JDNWF (**B**) in liver tissues based on ORA was presented and the pathways appearing in both sets of comparisons were shown in red (*P < 0.05). **C** After screening, 108 metabolites in the liver of ACLF rats were significantly altered by JDNWF. **D** Pathway analysis suggested citrate cycle, pyruvate metabolism and amino acid metabolism (arginine, citrulline and ornithine) were the potential pathways JDNWF regulated

Over-Representation Analysis on Wekemo Bioincloud was employed to find metabolic pathways significantly enriched. Metabolites with differences (T test, p < 0.05) between groups were analyzed. ACLF is mainly related to Ubiquinone and other terpenoid-quinone biosynthesis, Glutathione metabolism, TCA cycle, Amino acids metabolism. The therapeutic effect of JDNWF maybe related to TCA cycle, Amino acids metabolism, pentose phosphate pathway and fatty acids biosynthesis (Fig. 5A, B, Additional file 1: Table S2). In order to further obtain the differential metabolites between groups, metabolites in OPLS-DA model were screened. According to the condition of VIP > 1 and P < 0.05, 401 differential metabolites that contributed to the OPLS-DA model were screened, which were considered as representative metabolites of ACLF rats, and 361 metabolites representing the primary regulation of JDNWF were also screened. With the same conditions, we screened the differential metabolites between JDNWF and ACLF group. Finally, 108 metabolites altered by JDNWF were screened as biomarkers of JDNWF treatment, suggesting that these metabolites may play an important role in the metabolic pathway of JDNWF against ACLF (Fig. 5C, Additional file 1: Figure S4 and Table S3). Using MetaboAnalyst 5.0, the pathway analysis of these 108 metabolites revealed that the pathways altered by JDNWF were mainly focused on glucose metabolism (pyruvate, citrate) and amino acid metabolism (arginine, ctrulline and ornithine) (Fig. 5D and Additional file 1: Table S4).

### JDNWF contributed to driving the mitochondrial TCA cycle in ACLF rats

**Fig. 6:**
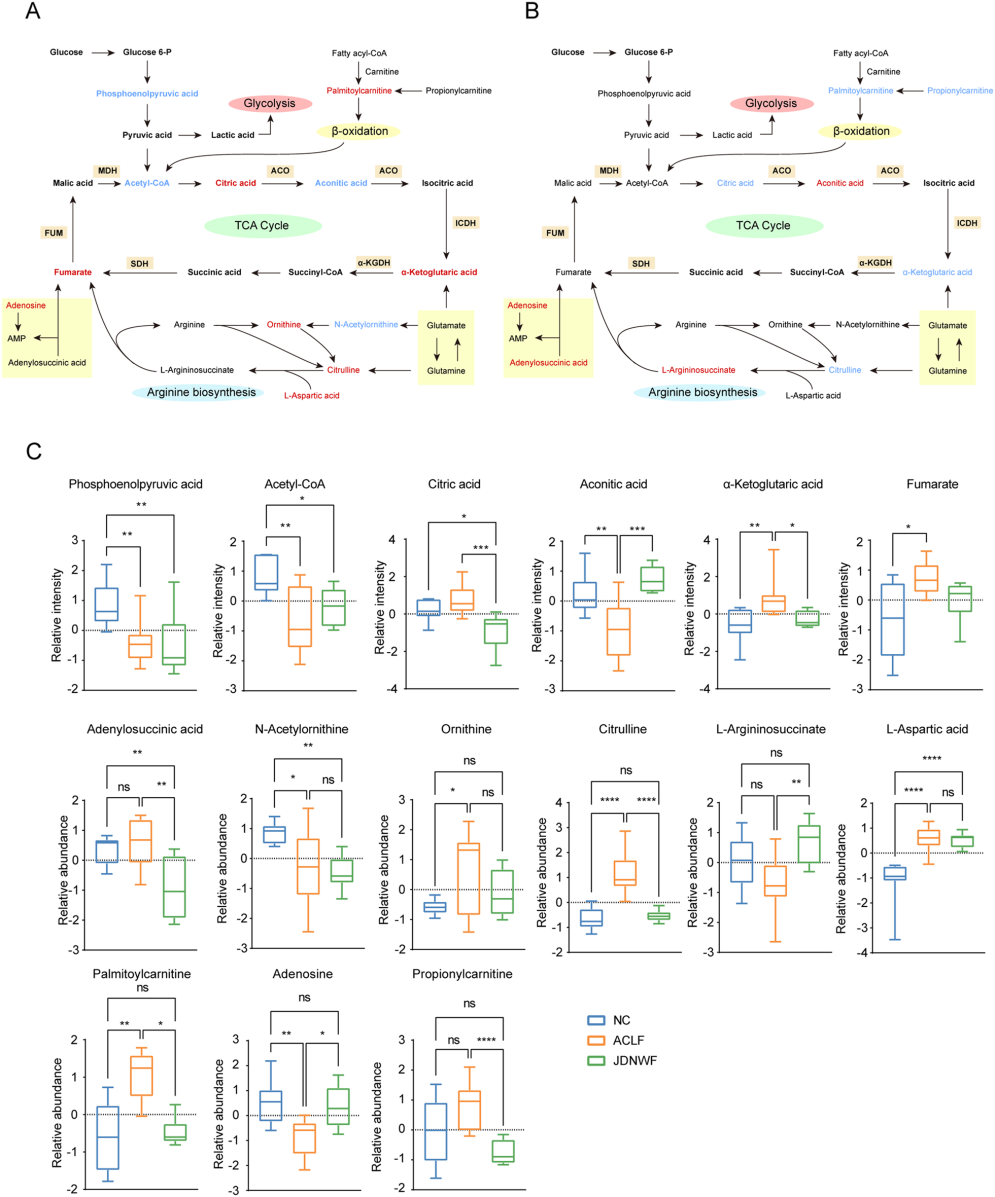
Metabolomics demonstrates the altered TCA cycle pathway in the liver of ACLF rats and the regulatory role of JDNWF. **A** Schematic diagram of the characteristic metabolic pathways in ACLF group and **B** JDNWF group. Red-labeled metabolites indicate that this metabolite was elevated in ACLF group or JDNWF group, while blue-labeled metabolites were decreased. **C** Relative levels of different metabolites in the abnormal metabolic pathways in the liver of ACLF rats. (*p < 0.05, **p < 0.01, ***p < 0.001, ****p < 0.0001)

As dysfunctional energy metabolism is a key risk factor for the progression of ACLF, altered relative abundance of intermediates in the TCA cycle were identified based on metabolomics (Fig. 6A and B). As a result, the relative intensity of α-ketoglutaric acid (α-KG) and fumarate were found to be elevated in ACLF rats, while phosphoenolpyruvic acid (PEP), acetyl-CoA, and aconitic acid were decreased. However, JDNWF reduced the relative intensity of α-KG, and citric acid, and restored the relative levels of aconitic acid (Fig. 6C). The metabolomic profile indicated that mitochondria-associated TCA cycle disorder was an intrinsic pathway of JDNWF in treating ACLF.

**Fig. 7:**
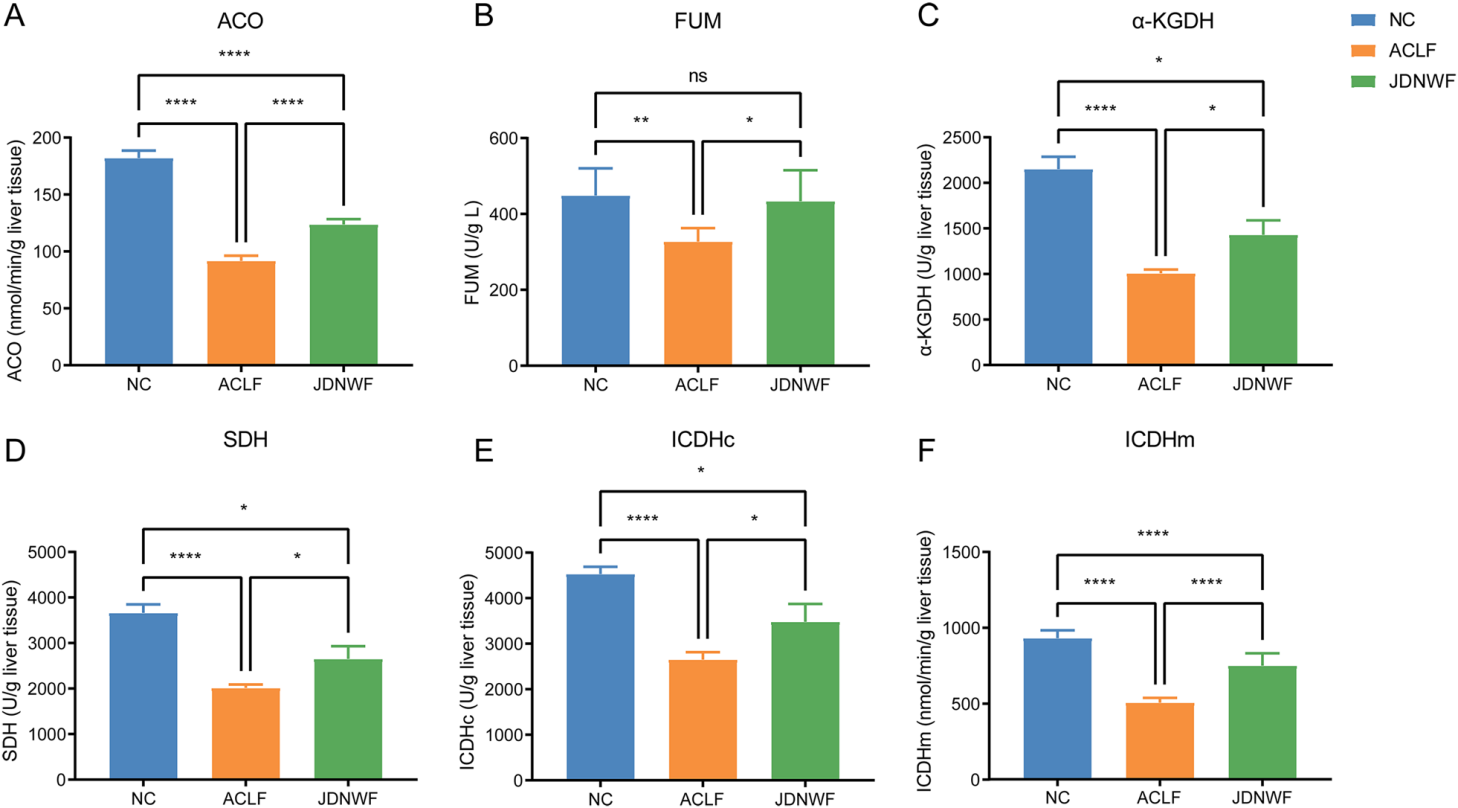
The activity of metabolic enzymes involved in the TCA cycle ACO (**A**), FUM (**B**), α-KGDH (**C**), SDH (**D**), ICDHc (**E**), ICDHm (**F**) (*P < 0.05, **P < 0.01, ****P < 0.0001)

The TCA cycle is driven by a series of mitochondrial enzymatic reactions, and the activity of these metabolic enzymes is essential for the maintenance of mitochondrial energy production. Thus, we further assayed the activities of selected metabolic enzymes and found that aconitase (ACO), isocitrate dehydrogenase (ICDH), α-ketoglutarate dehydrogenase (α-KGDH), fumarase (FUM), and succinate dehydrogenase (SDH) activities were all suppressed in ACLF rats. In contrast, JDNWF restored the activity of these enzymes to a certain extent (Fig. 7). Of these, ICDH and α-KGDH are involved in two irreversible reactions of the TCA cycle to ensure the priority availability of citric acid and fumaric acid, the two substrates of the TCA reaction. It was suggested that the activity of TCA cycle-reactive enzymes in ACLF is inhibited, causing intermediate metabolite accumulation and insufficient initiation substrate, which suggests a blockage of the tricarboxylic acid cycle pathway. JDNWF treatment drove the TCA cycle in ACLF rats by restoring the breakpoint in the TCA cycle.

### Mitochondrial damage in ACLF rats was ameliorated by JDNWF

**Fig. 8:**
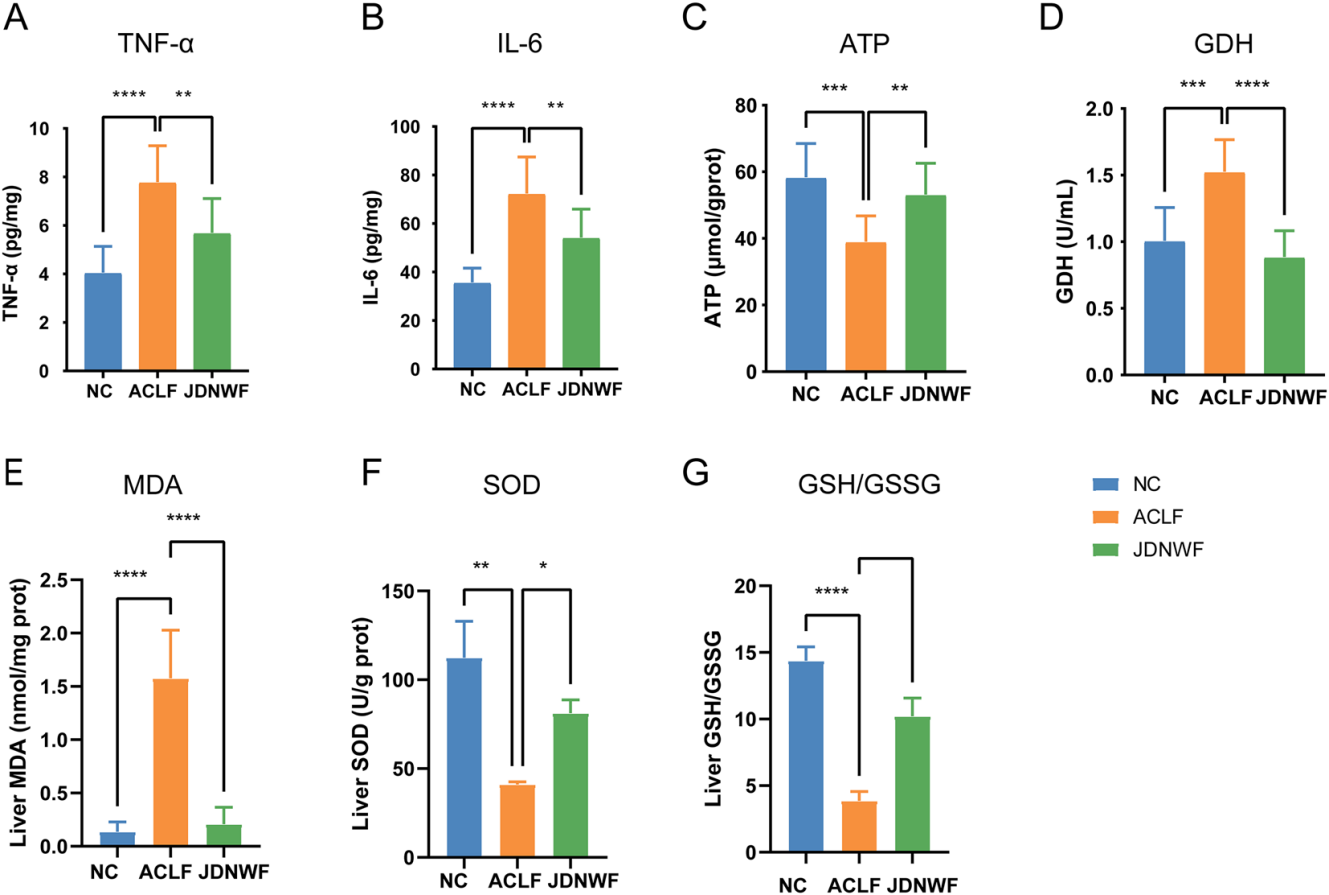
Effect of JDNWF on mitochondrial injury in ACLF rats. Levels of inflammatory factors TNF-α (**A**), IL-6 (**B**), and markers for mitochondrial injury marker ATP (**C**), GDH (**D**), and oxidative stress markers MDA (**E**), SOD (**F**), GSH/GSSG (**G**) were measured in rats (*P < 0.05, **P < 0.01, ***P < 0.001, ****P < 0.0001)

**Fig. 9:**
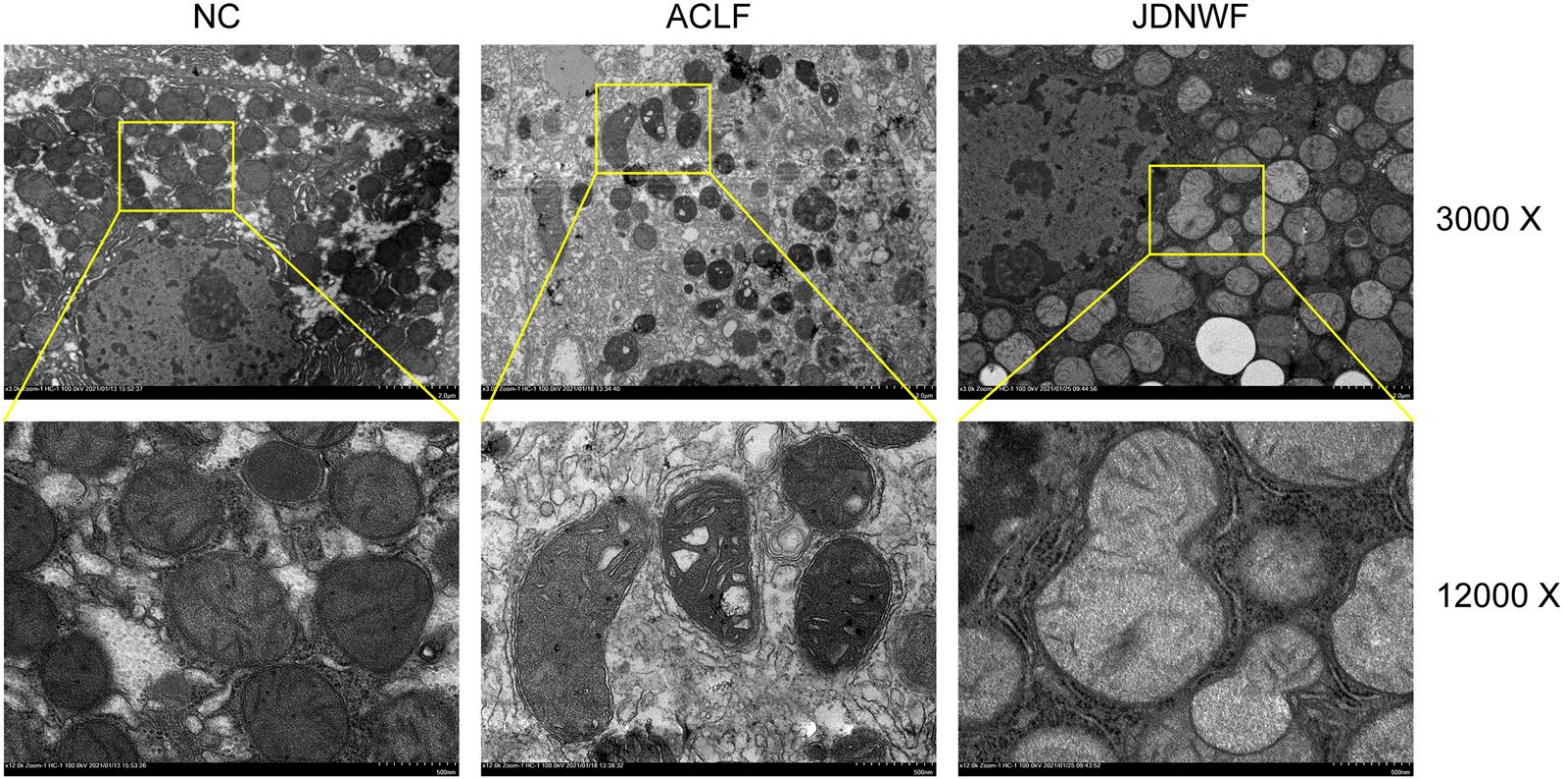
Significant damage was observed in the morphological structure of mitochondria in ACLF rats in TEM image

The TCA cycle is an essential pathway for energy production within the mitochondria. Mitochondria is the energy factory for the organism, producing more than 90% of the daily energy we required through oxidative phosphorylation (OXPHOS). In this study, the accumulation of metabolic intermediates in the TCA cycle and reduced metabolic enzyme activity reflected a mitochondrial dysfunction. In ACLF, inflammatory factors are closely related to mitochondrial dysfunction. For this purpose, we first measured the levels of inflammatory factors tumor necrosis factor-α (TNF-α) and interleukin 6 (IL-6) of rats and an increase of hepatic TNF-α, IL-6 was observed in ACLF rats. As expected, it was alleviated by JDNWF (Fig. 8A, B). Then, we detected serum GDH as well as liver ATP levels (markers of mitochondrial dysfunction) in each group. Serum levels of GDH were increased and liver ATP was decreased in ACLF rats, suggesting the presence of mitochondrial damage in ACLF rats while JDNWF treatment reduced GDH level and restored ATP level (Fig. 8C, D). Besides, we evaluated the oxidative stress level of rat liver tissue, which is often caused by mitochondrial damage. As a result, Malondialdehyde (MDA) level, a member of lipid peroxides, was increased in ACLF rat, while SOD and GSH/GSSG ratio, the antioxidant system of organism, were decreased. However, JDNWF reversed the levels of MAD, SOD, and GSH/GSSG ratio (Fig. 8E–G). We further observed the mitochondrial ultrastructural changes in each group by transmission electron microscopy (TEM) and found that the mitochondria of rat hepatocytes in the NC group were long and tubular in shape with well-integrated cristae and widely distributed in the cytoplasm. Morphological abnormalities were increased in the ACLF group, with swollen mitochondria, disrupted and poorly defined cristae, and vacuolation of the inner membrane (Fig. 9). However, alleviated mitochondrial injuries were presented in JDNWF group, suggesting JDNWF treatment contributes to the recovery of mitochondrial dysfunction.

### JDNWF regulated mitochondrial quality control (MQC) in ACLF rats

MQC is a critical mechanism for maintaining mitochondrial homeostasis, including mitophagy, mitochondrial biosynthesis and mitochondrial dynamics, hence we assayed the expression of proteins involved in MQC.

**Fig. 10:**
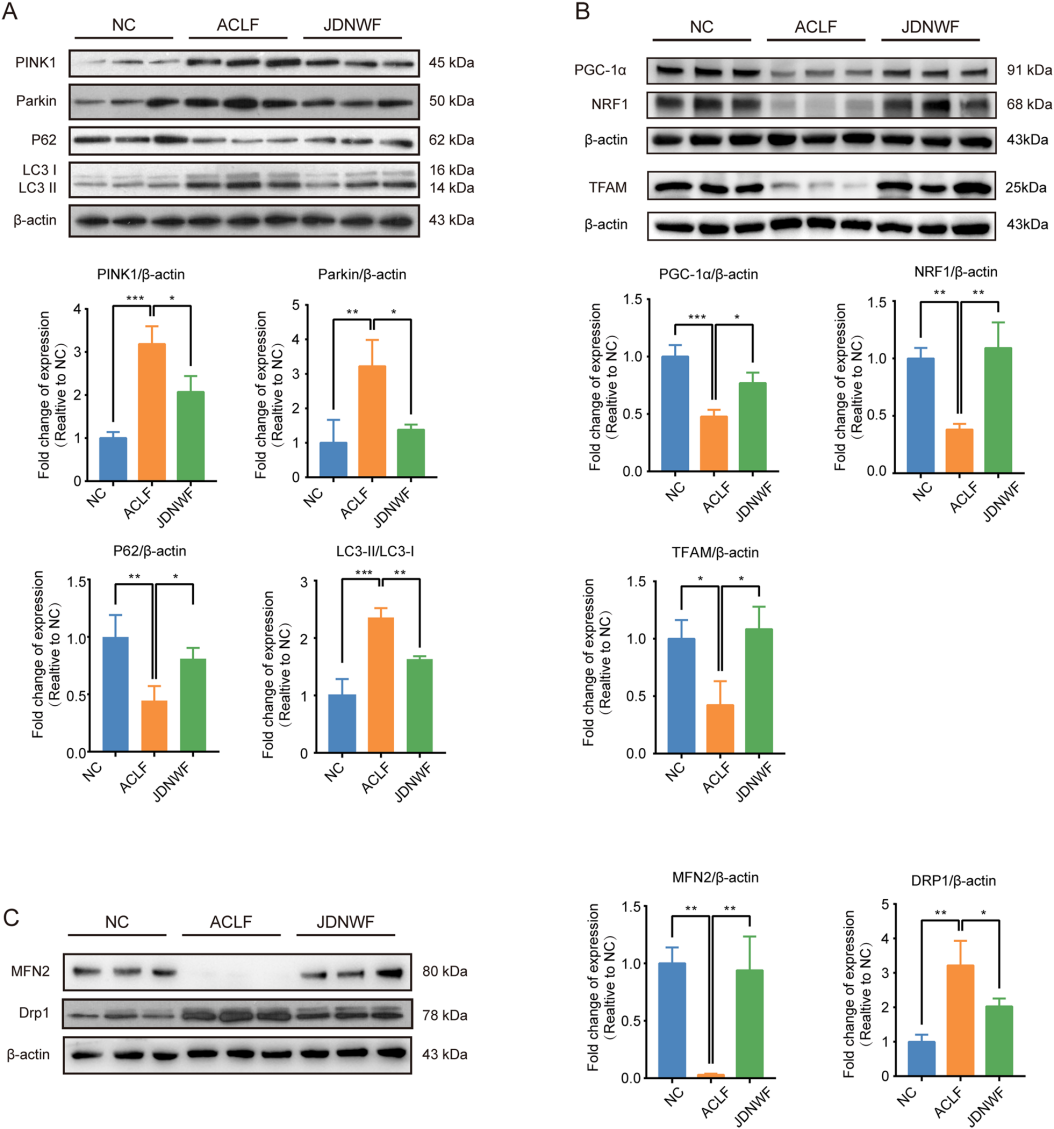
The effects of JDNWF on the protein levels of mitophagy markers PINK1, Parkin, P62, LC3 (**A**), and mitochondrial biosynthe-sis-related proteins PGC-1α, NRF1, and TFAM (**B**), and mitochondrial dynamics-related proteins MFN2 and Drp1 (**C**) were evaluated by Western blot. Data was normalized with β-actin (*P < 0.05, **P < 0.01, ***P < 0.001)

Firstly, it was found that the expression of the mitophagy signal PINK1 and Parkin was increased in the ACLF group compared to the NC group, while JDNWF attenuated such increases. Furthermore, the ratio of mitophagy marker LC3-II/LC3-I was significantly increased in ACLF rats, while the expression of autophagy substrate P62 was decreased. The levels of LC3-II /LC3-I were recovered in the JDNWF group (Fig. 10A).

We then assessed the expression of mitochondrial biosynthesis-related proteins. The expression of PGC-1α, NRF1, and TFAM was lower in ACLF rats than in the NC group, while JDNWF attenuated those decreases (Fig. 10B).

Finally, by measuring the expression of MFN2 and DRP1 to evaluate altered mitochondrial dynamics, we revealed that MFN2 levels were significantly reduced in ACLF rats and that JDNWF attenuated this reduction. In contrast, DRP1 levels were significantly increased in ACLF rats, and again JDNWF attenuated this effect (Fig. 10C).

**Fig. 11:**
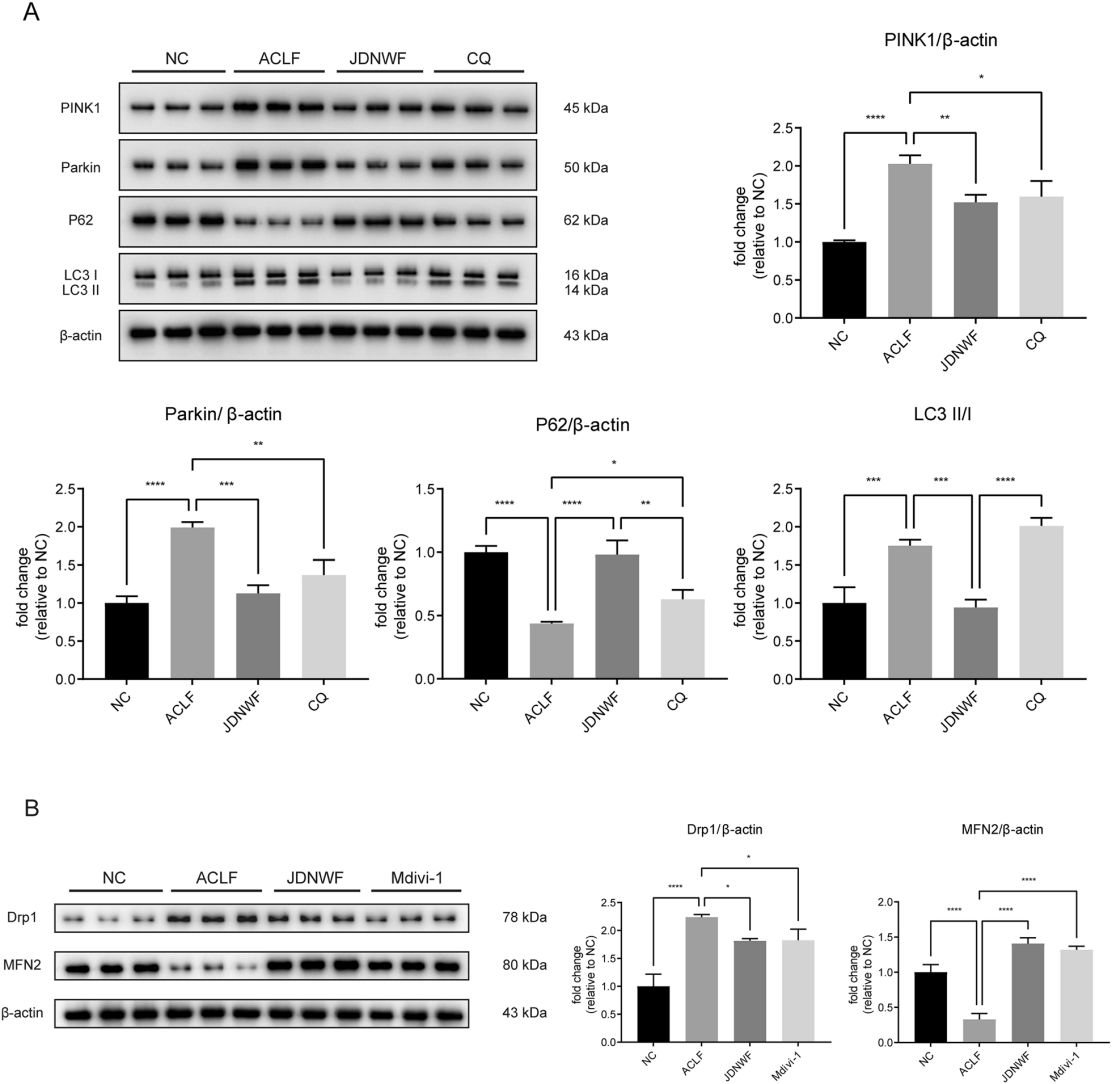
The levels of mitophagy markers PINK1, Parkin, P62, LC3 (**A**) and mitochondrial dynamics-related proteins MFN2 and Drp1 (**B**) were evaluated by Western blot after different interventions. Data was normalized with β-actin (*P < 0.05, **P < 0.01, ***P < 0.001, ****P < 0.001)

A mitophagy inhibitor, CQ, was used to establish the role of mitochondrial mitophagy in ACLF group. After CQ intervention, there was no significant change in the LC3-II/LC3-I ratio, while the level of the autophagy substrate P62 was significantly elevated, indicating that CQ effectively inhibited mitochondrial autophagy signaling (Fig. 11A). Besides, Mdivi-1, a mitochondrial division inhibitor, was employed. As a results, the expression of Drp1 in liver tissue of Mdivi-1 group was significantly lower than that of ACLF group, and the level of mitochondrial fusion protein MFN2 was significantly increased. The expression levels of Drp1 and MFN2 in JDNWF group were similar to those in the Mdivi-1 group, and there was no statistical difference, indicating that Mdivi-1 effectively inhibited the mitochondrial division in ACLF rats, while the JDNWF had a similar effect (Fig. 11B).

### JDNWF improves energy metabolism in ACLF rats through MQC

**Fig. 12:**
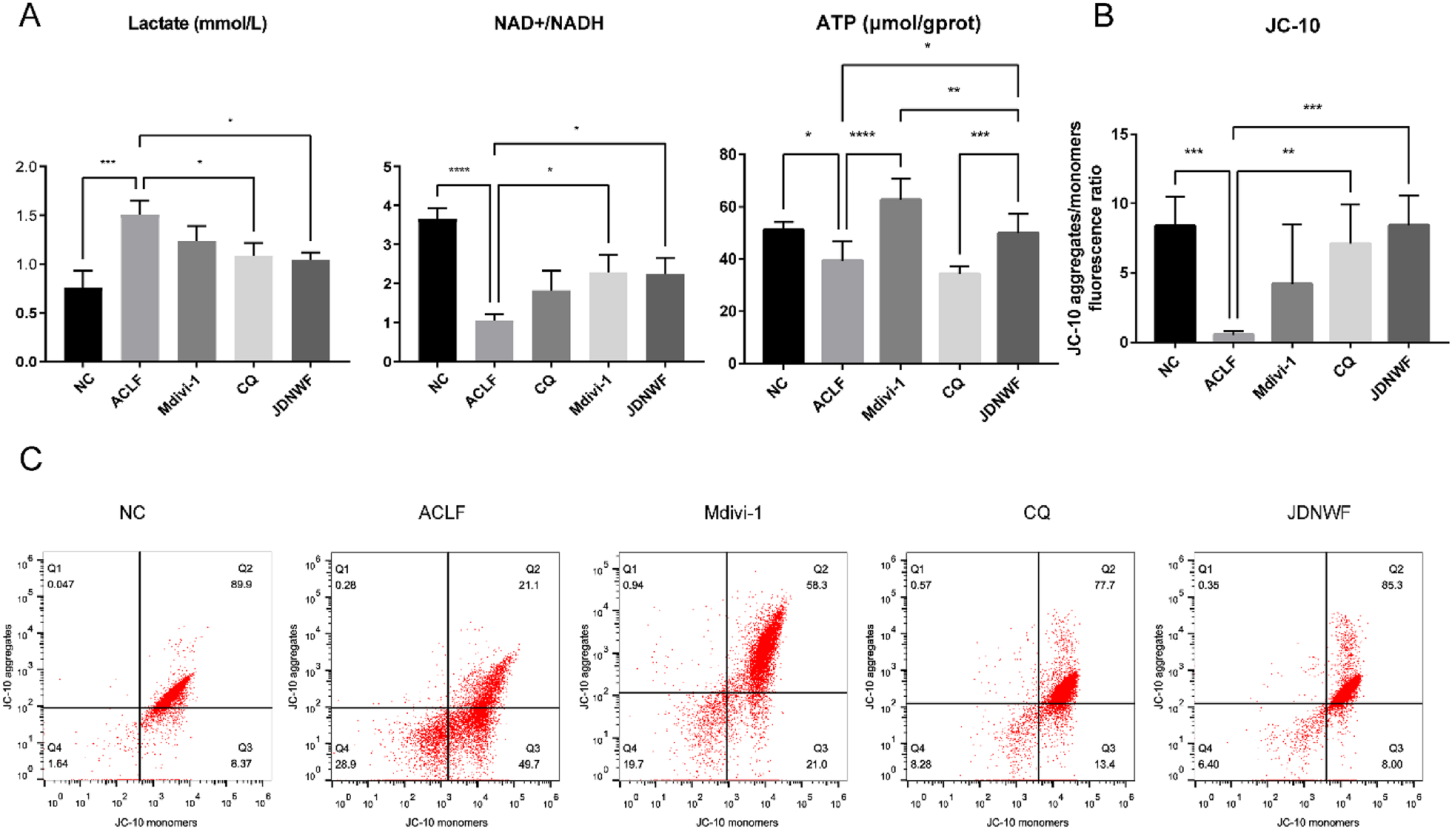
Effects of Mdivi-1, CQ and JDNWF on (**A**) serum lactate, hepatic NAD + /NADH, ATP levels and MMP (**B**) in ACLF rats were observed. MMP of liver tissue was detected by flow cytometry (**C**). (*P < 0.05, **P < 0.01, ***P < 0.001, ****P < 0.001)

To estimate the effects of JDNWF on mitochondrial function and energy metabolism in ACLF rats via MQC, serum lactate, liver NAD + /NADH and ATP were assayed in each group. The elevation of lactate and the decrease in NAD + /NADH are considered to be indicators of an active glycolytic process that reflects a decreased energy production. The decrease in ATP not only reflects mitochondrial damage, but also indicates a cellular energy deficiency. As a result, elevated serum lactate, lower NAD + /NADH and ATP levels were observed in ACLF rats while lactate, NAD + , and ATP were restored to a certain extent after JDNWF treatment. The treatments of CQ and Mdivi-1 reduced lactate and restored NAD + /NADH, respectively, in which mdivi-1 effectively restored ATP levels. However, CQ had no significant effect on the decreased ATP (Fig. 12A). Mitochondrial membrane potential (MMP) is an important parameter reflecting the function of mitochondria, for which a JC-10 staining was used to investigate the alteration of MMP. The results showed that liver MMP of ACLF rats was significantly decreased, whereas it was significantly up-regulated after the intervention of JDNWF and CQ. However, there was no significant change in the Mdivi-1 group (Fig. 12B and C).

## Discussion

ACLF is a severe disease with a high short-term mortality rate and there is an absence of effective treatment in modern medicine, except for liver transplantation. Recent studies have shown that the abnormal energy metabolism accompanied by mitochondrial dysfunction is closely associated with the severe inflammatory response and organ failure during ACLF [21, 22]. JDNWF is an effective empirical formula for ACLF, and our previous studies have demonstrated that this formula is able to inhibit hepatocyte apoptosis in ACLF rats to exert an anti-ACLF effect [6–8]. In this study, we first performed QC analysis of JDNWF and identified compounds as reported previously, such as quercetin, kaempferol, catalpol, notoginsenoside R1 and gallic acid [16]. Also, the protective effect of JDNWF against liver injury in ACLF rats was reconfirmed. However, the multi-component, multi-target, and multi-pathway characteristics of Chinese medicine make it challenging to explain the effects in a single pathway. Recently, metabolomics has been widely applied in explaining the biological processes of TCM in dealing with various diseases. Therefore, a metabolomic approach was adopted and our study presented the therapeutic effects of JDNWF in regulating mitochondrial damage and energy metabolism in rat liver during ACLF.

By analyzing the metabolic profiles of the liver tissues in each group of rats, TCA cycle was considered as the important pathway of JDNWF against ACLF. In ACLF rat, the activity of mitochondrial metabolic enzymes was significantly inhibited. In particular, ICDH and α-KGDH, their reduced activity largely causes the accumulation of the corresponding substrates, citric acid and fumaric acid, since the enzymatic reactions in which they are involved are irreversible. However, JDNWF not only improved the activity of these enzymes, but also restored the relative levels of aconitic acid, citric acid, and α-ketoglutaric acid, which may be associated with mitochondrial function.

TCA cycle is a central pathway of energy metabolism using carbohydrates, lipids and, amino acids as substrates. Acetyl CoA generated by the oxidation of pyruvate, fatty acids, and amino acids moves into the mitochondrial matrix and undergoes a series of reactions such as decarboxylation, dehydrogenation, and substrate phosphorylation. Finally, acetyl-CoA is oxidized to two CO_2_ molecules, along with three NADH molecules and one FADH2 molecule, which enter the mitochondrial respiratory chain to produce ATP through OXPHOS [23]. The TCA cycle is important for cellular homeostasis. This process is not only essential for energy supply but is even a major factor in influencing the response of the immune system and thus determining the outcome of the cells. Decreased levels of TCA cycle intermediates citric acid and aconitic acid were observed in LPS/D-GalN-induced ALF mice [24]. APAP-exposed HepG2 also showed reduced carbon sources entering the TCA cycle and up-regulated glycolysis and lactic acid production [25]. Interestingly, immune cells from ACLF patients suffer from reduced isocitric acid and succinic acid utilization, with two blocks in the TCA cycle, and patients' metabolic patterns were also more prone to glycolysis [21]. The results also showed that palmitoylcarnitine (carnitine attached by a long-chain fatty acid) accumulated in the liver of ACLF rats, while JDNWF effectively downregulated propionylcarnitine, an indicator of impaired fatty acid metabolism, and decreased palmitoylcarnitine. It was suggested that JDNWF may promote the transportation of fatty acyl-carnitine to mitochondria for β-oxidation and reduction to acetyl-CoA to eventually provide energy with the form of ATP.

Recently, the pathogenesis of ACLF has been discussed and it is believed that ACLF is related to the change of energy metabolism under the background of systemic inflammation [1]. The CANONIC study from EASL-CLIF indicated that mitochondrial dysfunction and pathological alterations in energy metabolic pathways are present in ACLF patients [22]: on the one hand, the high energy cost of the inflammatory response leads to an insufficient energetic supply to hepatocytes; on the other hand, the mitochondrial dysfunction caused by the severe inflammatory response greatly restricts the progress of mitochondrial metabolism: TCA cycle, OXPHOS, and β-oxidation [1, 22, 26], thus reducing ATP synthesis and promoting the development of ACLF. In this study, it was reflected a disorder in the TCA cycle and a decrease in the utilization of α-ketoglutarate and fumarate. However, JDNWF helps to restore the breakpoint in the TCA cycle and drive mitochondrial metabolism in ACLF.

In the present study, Arginine biosynthesis was a critical metabolic pathway and was closely related to the TCA cycle. Citrulline is synthesized from ornithine via the urea cycle, followed by a synthesis of arginine in the presence of Argininosaccinate Synthase1 (ASS1). However, hyperornithine and hypercitrulline are associated with hyperammonemia and mitochondrial damage. Interestingly, mitochondrial dysfunction and hyperammonia are pathological and metabolic features of hepatocytes in ACLF patients [22, 27]. As a consequence of mitochondrial damage, ornithine cannot be imported into the mitochondria, and causes impaired urea circulation. The deficiency of ornithine aminotransferase in mitochondria eventually leads to the accumulation of citrulline, while high ornithine and citrulline reduce GSH levels further triggering mitochondrial damage [28].

Since mitochondria play a central role in the TCA cycle, mitochondrial dysfunction is a major factor in the development of ACLF. In this study, serum GDH increased and liver ATP decreased in ACLF rat. GDH is a mitochondrial matrix enzyme and an increase of serum GDH is a marker of mitochondrial damage [29]. What’s more, oxidative stress was also triggered in the rat liver during ACLF. After JDNWF treatment, mitochondrial damage and oxidative stress were attenuated in rat liver. Mitochondria maintain a protection system in response to injury- Mitochondrial Quality Control (MQC). Mitochondria carry out continuous mitochondrial fission and fusion, mitochondrial biosynthesis and mitophagy in response to constantly changing cellular states and tissue environments, thereby maintaining a relatively stable state of mitochondrial morphology, quantity, and quality [30–32].

Mitochondrial biosynthesis is a process of cell synthesis through nuclear and mitochondrial genomes, resulting in the generation of new mitochondria. New mitochondria restore and enhance cellular OXPHOS, ATP synthesis activity, and repair mitochondria functions [33]. Peroxisome proliferator-activated receptor gamma coactivator 1α (PGC-1α) is the first stimulator of mitochondrial biogenesis. By activating the intermediate transcription factor NRF1, PGC-1α regulates the expression of mitochondrial respiratory chain complex enzyme, and then stimulate the final synthesis effector TFAM [34, 35]. Studies have demonstrated that the administration of mitochondrial biosynthesis inducers, which activate PGC-1α and NRF1, can reduce APAP-induced liver injury [36]. In the present study, JDNWF was effective in restoring reduced levels of PGC-1α, NRF1, and TFAM in ACLF rats, indicating that JDNWF improved mitochondrial biosynthesis.

Mitochondria keep performing fusion/fission to maintain the homeostasis of its network structure to adapt to the changing environment [37]. Mitochondrial fission contributes to the reduction of damaged/dysfunctional mitochondria by segmenting the mitochondrial network, thereby removing functionally impaired mitochondria through mitophagy; Mitochondrial fusion is the process of fusing into a single mitochondrion, maintaining the relative integrity of the individual mitochondria to alleviate structural and functional defects. Mitochondrial fusion and division imbalance will lead to cellular dysfunction and cell death [38]. Dynamin-related protein 1 (DRP1) can be recruited to the mitochondrial membrane to induce mitochondrial fission, while mitochondrial fusion protein 2 (Mitofusin-2, Mfn2) can be embedded in the outer membrane of two mitochondria to mediate mitochondrial fusion and thus restore mitochondrial integrity [29–31, 39]. Mdivi‐1, a mitochondrial fission inhibitor, prevents mitochondrial fission and increased liver NAD + /NADH ratio and ATP content in ACLF rats in our study. Our findings also suggest that JDNWF can reduce mitochondrial fission and promote mitochondrial fusion to regulate mitochondrial dynamics.

Mitophagy completes the degradation of damaged mitochondria by specifically coating the functionally impaired mitochondria to form autophagosomes. The degradation products obtained are used to achieve the recycling of components and renewal of mitochondria [40, 41]. The PINK1/Parkin signaling pathway is a widely studied mitophagy pathway. PTEN induced putative kinase 1 (PINK1) is stabilized on the outer membrane of damaged mitochondria and subsequently recruits Parkin and ubiquitin [42, 43], to interact with mitochondrial autophagic substrate protein P62, thus attracting LC3 to form autophagosomes and promoting mitophagy [44–46]. However, studies have suggested that excessive mitophagy leads to redundant mitochondrial degradation and eventual cell damage. That is, when new mitochondrial synthesis is insufficient and the coupled processes of mitochondrial fission and mitochondrial mitophagy are excessive, mitochondria will fail to restore homeostasis, leading to cellular dysfunction [30, 47, 48]. CQ, an inhibitor of lysosomal acidification, significantly increased p62 protein accumulation. Accordingly, CQ intervention improved lactate accumulation and restored mitochondrial membrane potential in ACLF rats. Besides, our results suggested that JDNWF prevents excessive mitophagy in ACLF rats, and together with the discussion above, it was concluded that JDNWF ameliorates mitochondrial dysfunction in ACLF rats by regulating MQC.

In similarity to reported studies, our research showed a significant mitochondrial damage and energy metabolism disorder in the liver of ACLF rats. JDNWF is probably to improve mitochondrial function via the MQC pathway, thus restoring energy production in the liver. The metabolomics approach provides direction for our study, suggesting that measures targeting mitochondrial function and energy metabolism are effective therapies for ACLF as well. Certainly, it was a predictive result. Therefore, further pharmacological experiment is necessary.

## Conclusions

In this study, the potential mechanism of JDNWF for ACLF was analyzed by untargeted metabolomics with LC–MS/MS. Energy metabolism might be the metabolic pathway for JDNWF to improve ACLF, which is associated with mitochondrial dysfunction. Further studies suggested that JDNWF may prevent ACLF by improving dysregulation of MQC. Our study demonstrates that metabolomics is an effective method for evaluating TCM therapies, and to a certain extent reflects its multi-pathway and multi-target characteristics. In the future, other molecular mechanisms will be more fully explored to uncover the multiple mechanisms of JDNWF for ACLF.

### Supplementary Information

**Additional file 1: Figure S1.** Results of the UPLC-MS/MS analysis of the JDNWF. **Figure S2.** PCA analysis of quality control samples. **A** PCA diagram of all samples. **B** Distribution of PC1 in each sample. **Figure S3.** The typical total ion current (TIC) chromatogram of metabolites generated by LC-MS/MS. **Figure S4.** Cluster heat map of 108 significant metabolites. **Table S1.** Identification of compounds in JDNWF. **Table S2.** Top 20 metabolic pathways in rat liver analyzed by ORA. **Table S3.** 108 significant metabolites of JDNWF in the treatment of ACLF. **Table S4.** Pathway analysis of 108 significant metabolites.

## Data Availability

All processed data for the study are included within the manuscript. Raw datasets are available from the corresponding author on reasonable request.

## Abbreviations

ACLF: Acute-on-chronic liver failure
ALB: Albumin
ALT: Alanine aminotransferase
APTT: Activated partial thromboplastin time
AST: Aspartate aminotransferase
GDH: Glutamate dehydrogenase
IL-6: Interleukin-6
INR: International normalized ratio
JDNWF: Jieduan-Niwan formula
MQC: Mitochondrial quality control
OXPHOS: Oxidative phosphorylation
PT: Prothrombin time
TBiL: Total bilirubin
TCA cycle: Citrate acid cycle
TNF-α: Tumor necrosis factor-α
