# Supplementary figures and images for: Liver metabolomics reveals potential mechanism of Jieduan-Niwan formula against acute-on-chronic liver failure (ACLF) by improving mitochondrial damage and TCA cycle
Source: Chin Med. 2023 Nov 30;18:157. doi: 10.1186/s13020-023-00858-x (PMC10691013; doi:10.1186/s13020-023-00858-x)

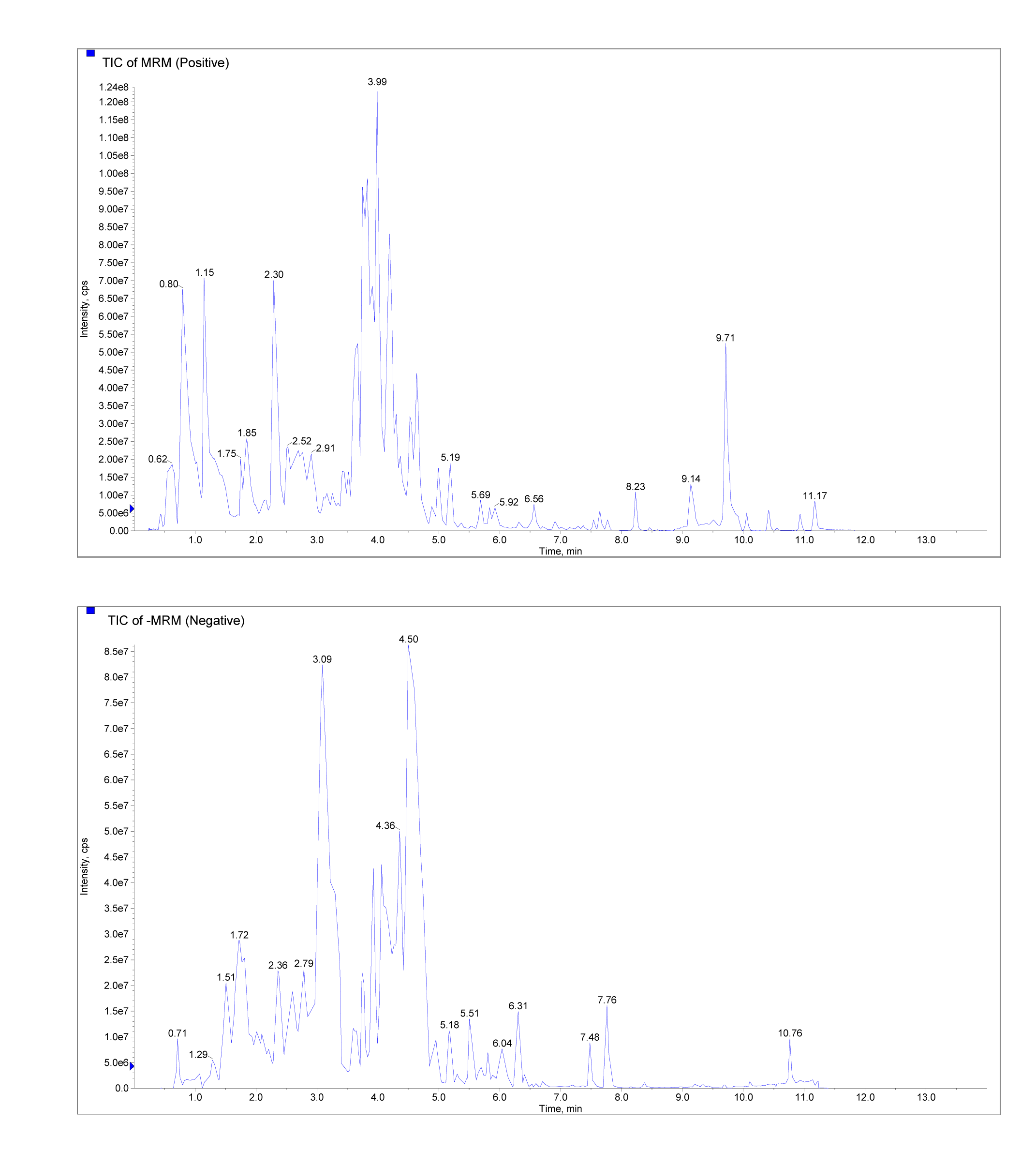

Supplement: Supplementary file 1 — Additional file 1: Figure S1. Results of the UPLC-MS/MS analysis of the JDNWF. Figure S2. PCA analysis of quality control samples. A PCA diagram of all samples. B Distribution of PC1 in each sample. Figure S3. The typical total ion current (TIC) chromatogram of metabolites generated by LC-MS/MS. Figure S4. Cluster heat map of 108 significant metabolites. Table S1. Identification of compounds in JDNWF. Table S2. Top 20 metabolic pathways in rat liver analyzed by ORA. Table S3. 108 significant metabolites of JDNWF in the treatment of ACLF. Table S4. Pathway analysis of 108 significant metabolites. [file 13020_2023_858_MOESM1_ESM.zip › Supplementary/Figure S1.tif]

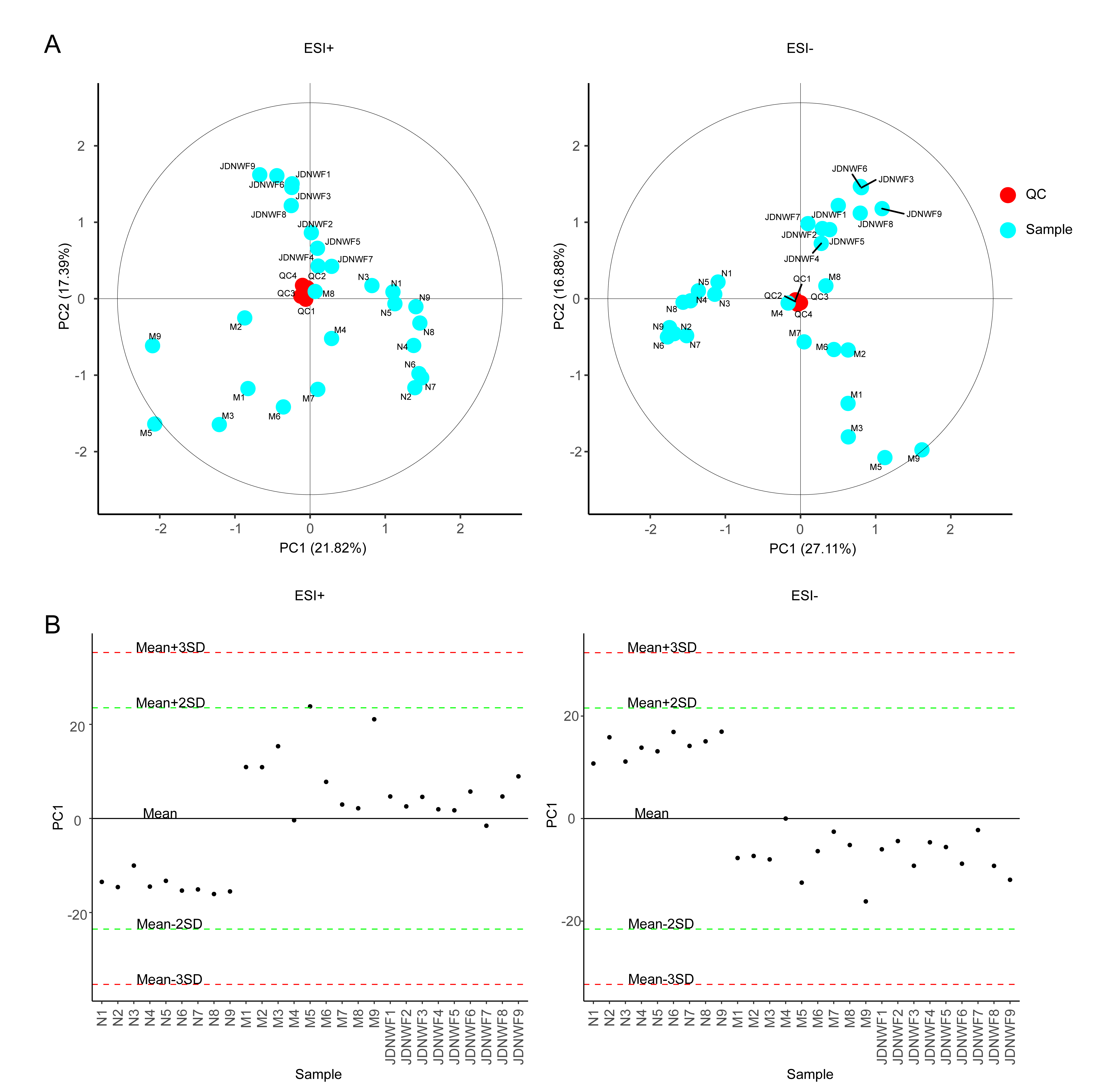

Supplement: Supplementary file 1 — Additional file 1: Figure S1. Results of the UPLC-MS/MS analysis of the JDNWF. Figure S2. PCA analysis of quality control samples. A PCA diagram of all samples. B Distribution of PC1 in each sample. Figure S3. The typical total ion current (TIC) chromatogram of metabolites generated by LC-MS/MS. Figure S4. Cluster heat map of 108 significant metabolites. Table S1. Identification of compounds in JDNWF. Table S2. Top 20 metabolic pathways in rat liver analyzed by ORA. Table S3. 108 significant metabolites of JDNWF in the treatment of ACLF. Table S4. Pathway analysis of 108 significant metabolites. [file 13020_2023_858_MOESM1_ESM.zip › Supplementary/Figure S2.tif]
